# Sixty-month comperative evaluation of a glass hybrid restorative and a composite resin in non-carious cervical lesions of bruxist individuals

**DOI:** 10.1007/s00784-024-05570-0

**Published:** 2024-03-09

**Authors:** Uzay Koc Vural, Ece Meral, Esra Ergin, Sevil Gurgan

**Affiliations:** Hacettepe University, Ankara, Turkey

**Keywords:** Non-carious cervical lesions, Bruxism, Glass hybrid restorative system, Nano-ceramic composite resin

## Abstract

**Objective:**

To compare the clinical performance of a glass hybrid (GH) restorative and a nano-ceramic composite resin (CR) in the restoration of non-carious cervical lesions (NCCLs) of bruxist individuals in a 60-month randomized clinical trial.

**Materials and methods:**

Twenty-five bruxist candidates having NCCLs were recruited in this clinical study. The depth, height (cervico–incisal), width (mesio-distal), internal angles of the NCCLs, degree of tooth wear (TWI) and gingival index (GI) were measured. One hundred-and-forty-eight NCCLs were restored either with a GH restorative (Equia Forte Fil) or a CR (Ceram.X One Universal). Modified USPHS criteria was used to evaluate restorations after 1 week and 12, 24, 36 and 60 months. Pearson’s Chi-Square, Fisher’s Exact and Cochran Q tests were run for analysis. Survival rates of the restorations were compared with Kaplan–Meier analysis (*p* < 0.05).

**Results:**

After 60 months, 97 restorations in 15 patients were examined. The recall rate was 60.0%. Retention rates were 73.5% for CR and 66.7% for GH. A total of 29 restorations were lost (13CR (26.5%), 16GH (33.3%)). There was not a significant difference between tested restoratives in retention (*p* = 0.464), marginal adaptation (*p* = 0.856) and marginal discoloration (*p* = 0.273). There was no relationship between internal angle, depth, height or width and retention of the GH or CR restorations (*p* > 0.05). The increase in retention loss and marginal discoloration of both restorations over time were significant (*p* < 0.001). Sensitivity or secondary caries were not detected after 60 months.

**Conclusion:**

GH and nano-ceramic CR showed similar clinical performances in NCCLs after 60 months in patients with bruxism.

**Clinical significance:**

After 60 months, CR and GH materials showed clinically acceptable performances in restoration of NCCLs in patients with bruxism.

## Introduction

Parafunctional habits can affect the oral health and longevity of restorations. Bruxism is one of the most prevalent habits which is defined as the diurnal (awake bruxism) or nocturnal (sleep bruxism) repetitive jaw-muscle activities, including clenching, grinding, and gnashing of teeth [1, 2]. The main cause of bruxism remains undetermined, though it is believed to be multifactorial [3, 4]. Researchers usually associate awake bruxism with emotional stress whereas sleep bruxism seems to comorbid with some sleep disorders such as; parasomnias, obstructive sleep apnea and restless leg syndrome [2, 5]. Although several methods are suggested to overcome this problem, none of them can permanently treat bruxism yet [6]. Therefore, if a patient has bruxism, this condition usually remains chronic. Due to the continually increased masticatory muscle activity and occlusal overloads, bruxism can lead to clinical complications such as tooth tissue destructions, loss, or fracture of dental restorations, and temporomandibular disorders [7].

NCCLs are multifactorial pathological processes characterized by dental hard tissue loss at the cementoenamel junction, that occurs regardless of bacterial processes [8]. These lesions are directly related with the location, length, frequency, and intensity of the occlusal stresses. If not restored properly, NCCLs may cause troubling consequences ranging from dentin hypersensitivity, caries development, restoration loss to root fracture [9]. Previous research had associated bruxism with increased prevalence of NCCLs [10–12] and restoration failures [13–15]. Therefore, it is very important to find out the best restorative material alternatives for NCCLs in the presence of bruxism.

The material of choice for the restoration of NCCLs are currently glass ionomer cements (GIC) and composite resins (CR) [16]. CRs possess some advantages such as high aesthetic and mechanical properties, however, these materials have some challenges for adhesion to dentin substrate in NCCLs [17, 18]. GICs are the other favoured restorative material options for the restoration of NCCLs. Beside their advantages such as chemical adhesion, biocompatibility, fluoride release and dentin-like elastisity modulus; still there are some contraversies about their lower aesthetic and mechanical properties that can adversely affect the long term success of the restorations [19].

For the purpose of overcoming the drawbacks of these materials, a number of new materials have been released from both resin composite and glass ionomer fronts. A relatively new generation, Glass Hybrid (GH) restoratives are reinforced GIC materials that are obtained by incorporation of smaller, more reactive silicate particles to the powder and higher molecular weight acrylic acid molecules to the liquid [20, 21]. As a result, a material with superior mechanical properties was formed and when used with its nano-filled resin coating, it can lead to more aesthetic and wear resistant restorations [22]. Newer CRs with enhanced physical and mechanical characteristics have also become available recently. Nano-CRs, which include nano-fillers ranged between 0.005–0.01 nm [23], claim to have better aesthetics along with better fracture and wear resistance, higher compressive and diametral tensile strength, minimal polymerization shrinkage [24].

Although there are several methods to test the performance of dental materials, the most reliable data can only be delivered from the long term clinical trials. Thus, current study aims to compare the mid-long term clinical performance of a GH restorative (Equia Forte Fil) and a CR (Ceram.X One Universal) in NCCLs of bruxist individuals after 60 months. The tested null hypothesis was that the clinical performances of the tested GH restorative and nano-ceramic CR would be similar.

## Materials and methods

### Sample size calculation

To obtain a sufficiently large sample size of restorations and thus to enhance the power of this study, total sample size was calculated using the G-Power package. Chi-square tests revealed minimum number of restorations as 62 per group to determine the ƒ = 0.5 effect difference between the study groups. Predetermined parameters were: power = 90%, alpha error = 5%, and assumption of DF = 4. Considering drop-outs and study design, it was decided to perform 74 restorations for each group.

### Study design

This study was designed as a single-center, split-mouth, double-blind and randomized controlled clinical study carried on patients having bruxism. Ethical considerations were addressed by confirmation of the study protocol by the local ethics committee of the university under number KA-16020. The study was also registered to ClinicalTrials.gov with the ID NCT03713827. Non-hospitalized bruxist patients meeting the inclusion criteria with at least two NCCLs situated on the buccal surfaces of the teeth and self-reported chronic bruxism condition with coexistent clinical symptoms of bruxism were included in this study. The teeth were included if they had occlusal contact. The patients were excluded from the study if they had; 1) chronic periodontitis, 2) intensive medical history (severe systemic diseases or disabilities that may prevent them from attending to the recalls e.g., severe diabetes, cancers, cardiovascular diseases, etc.), 3) unacceptable oral hygiene (oral hygiene index > 0 indicating any visible plaque at any tooth [25] and/or inflammation on adjacent gingiva (Gingival Index > 0 indicating bleeding on probing at any tooth) [26], 4) high caries activity (3 or more new caries lesions within the last 36 months) and if their NCCLs were previously restored, on mobile teeth or in contact with removable dentures. The assessment of high caries activity was based on recent dental examinations, patients' previous radiographs and their self-reports. Since all patients received their previous dental treatments at our University Hospital, their previous dental records and history was checked. Only the lesions requiring treatment were qualified as new caries [27, 28]**.** Twenty-five patients with the mean age of 55 ± 8.3 of years old (ranged between 42–72 years), meeting previously described inclusion criteria were enrolled in this study. After clearly explaining the objective, clinical procedure and study-related risks, written consents of the patients were obtained.

The diagnosis of bruxism was made according to non­instrumental approaches [26] including a combination of clinical examination, patient history, and self-reported symptoms related to bruxism and digital photos were obtained by a digital camera (Canon, DS126311, Canon inc.,Taichung,Taiwan) to ensure the pretreatment conditions (clinical signs of bruxism including occlusal wear facets, craze lines and fractures). The patients were asked for awareness of tooth-grinding or tooth-clenching during sleep and/or awake. The individuals who displayed at least one affirmative response to the questions (Table 1, Section A), had a minimum of two positive clinical signs of bruxism (Table 1, Section B) and at least one self-reported symptom (Table 1, Section C) at the same time were classified as bruxists. [29–31].

**Table 1:** Diagnostic criteria of bruxism [29–31]

A) At least one affirmative response to following questions: 1) Have you ever been told you grind teeth at night while sleeping? 2) Have you grind your teeth anytime of the day?
B) The presence of at least two of the following clinical signs are present: 1) Occlusal wear facets, 2) Craze lines and/or fractures 3) Masseter muscle hypertrophy upon voluntary forceful clenching
C) The presence of at least one of the following self-reported symptoms: 1) Jaw muscle discomfort, fatigue, or pain and jaw lock upon awakening 2) Earache and/or temporal headache

The margins of all the lesions were coronally in enamel and cervically in dentin. The characteristics of the lesions regarding the shape (wedge, sharp, saucer or rounded), dimensions (depth, cervico-incisal height and mesio-distal length), internal angle (1: 0–45°, 2: 45–90°, 3:90–135°, 4: 135–180°) [32], tooth wear index (TWI) (0: No loss of enamel surface characteristics, 1: Loss of enamel surface characteristics, 2: Loss of enamel exposing dentine for less than one third of surface, 3: Loss of enamel exposing dentine for more than one third of surface, 4: Complete enamel loss–pulp exposure secondary dentine exposure) [33] and gingival index (GI) (0: No bleeding, 1: Bleeding some seconds after probing, 2: Bleeding immediately after probing, 3: Bleeding on probing spreading towards the marginal gingiva) [34] were recorded. Measurements were performed using a CPI probe (CPI, WHO 973/80 Martin, Solingen, Germany).

### Randomization

The patients were blinded about the group assignments. For each patient, half of the lesions were treated with a GH restorative (Equia Forte Fil, GC, Tokyo, Japan) and the other half were treated with a CR (Ceram.X One Universal, Dentsply, DeTrey, Konstanz, Germany), randomly. The restorative material for the first lesion was determined by a coin toss and the other material was used to restore the second lesion. In the situation when the patient has more than 2 lesions, the teeth were restored according to the following order; upper right, upper left, lower left, and lower right quadrants.

### Treatment protocol

**Fig. 1:**
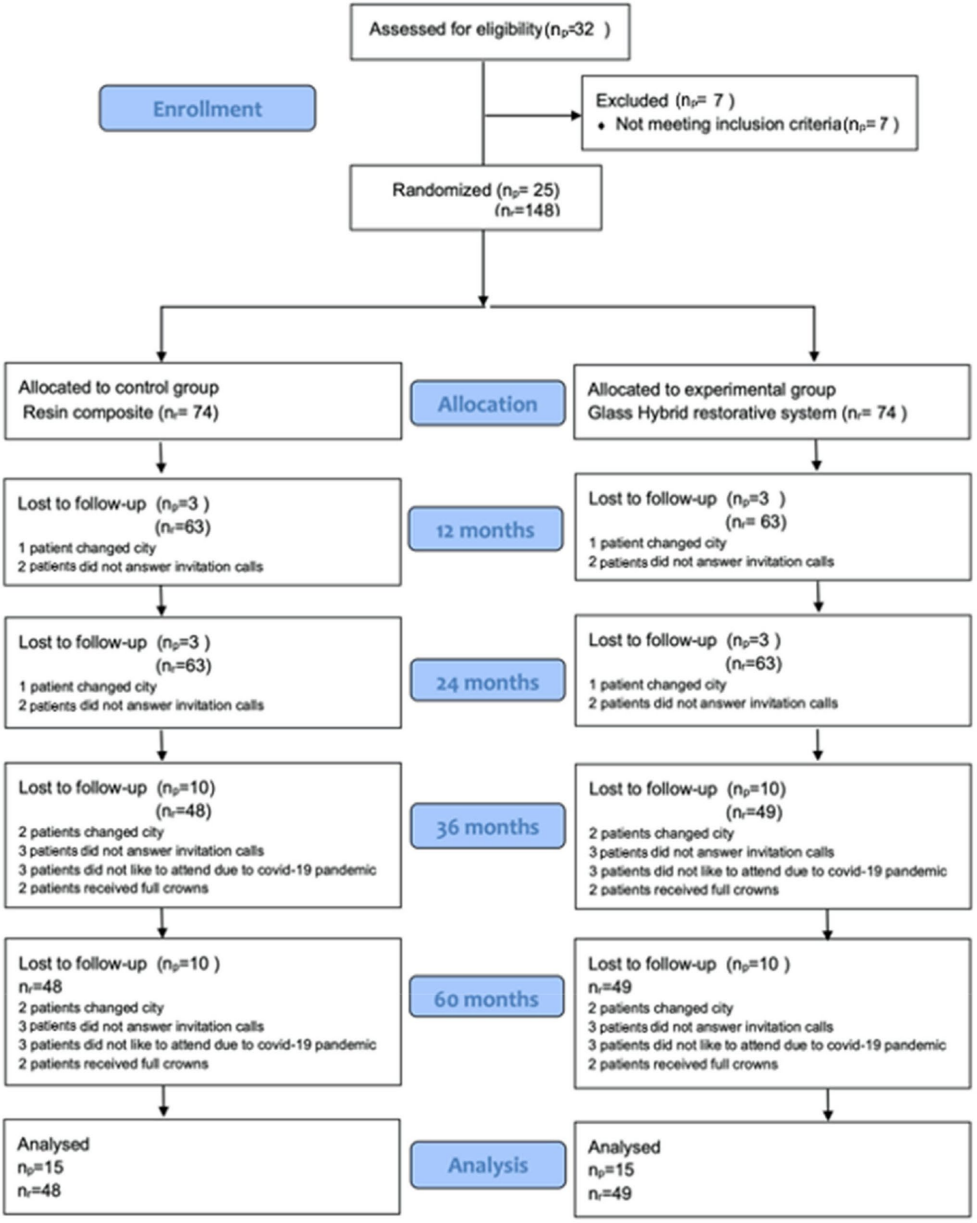
CONSORT flow diagram

The materials used and the flow diagram of the study are shown in Table 2 and Fig. 1.

**Table 2:** Applied materials and their compositions according to the manufacturers’ information

Material	Manufacturer	Composition
Equia Forte Fil	GC, Tokyo, Japan	Powder: 95% strontium fluoroalumino-silicate glass, 5% polyacrilic acid. Liquid: 40% aqueous polyacrylic acid
Equia Forte Coat	GC, Tokyo, Japan	50% Methyl methacrylate, 0.09% camphorquinone
Ceram.X One Universal	DENTSPLY DeTrey Konstanz, Germany	Matrix: Methacrylate-modified polysiloxane, polyurethane methacrylate, Bis-EMA, TEGDMA, Fillers: 77–79% by weight nanofillers and organically modified nano- ceramic particles, Barium glass and Ytterbium fluoride, Camphorquinone, pigments
Prime & Bond Elect One	DENTSPLY DeTrey Konstanz, Germany	Bifunctional acrylate, Acidic acrylate, Functionalized phosphoric acid ester, Water, Tertiary butanol, Initiator, Stabilizer
Etching gel	GC, Tokyo, Japan	37% phosphoric acid

Operative procedures were performed by a single experienced investigator to minimize variations. Patients were enrolled in March 2016. The operator applied both test materials to 20 patients who were not part of the main study in April 2016 and the selected patients were treated between May to August 2016. Two previously calibrated evaluators assessed the restorations and the operator was considered calibrated when each restoration was scored as Alpha to perform restorative procedures throughout the study.

The treatment protocol started with the oral hygiene instructions followed by cleaning of the teeth using a polishing brush and slurry of pumice. The shade selection of both materials was performed according to the shade guide of the relevant material. Cotton rolls and saliva ejectors were used for isolation. Teflon tape was used to isolate the treated tooth from its neighboring teeth during the etching, bonding and restoration processes. An equal number of restorations with each material were performed on all patients. The restorations were done without any enamel bevel preparation either with a GH or a nano-ceramic CR according to their manufacturers’ instructions as follows;

- **GH restoration (*n* = 74)**: The capsule of the GH restorative material (Equia Forte Fil) was shaken and activated by pushing the plunger prior to mixing for 10 s by a mixing device (Softly, Satelec Acteon, Merignac Cedex, France). The prepared material was then immediately injected to the lesion by the help of capsule applier and a hand instrument was used for contouring. After 2.5 min from the starting of the mixing, the finishing and polishing procedure were performed by using ultrafine diamond burs (Diatech Dental AC, Heerburgg, Switzerland) and abrasive discs (Sof-Lex Pop-On discs, 3 M ESPE, St Paul, MN, USA) and followed by the application of coating agent (Equia Forte Coat) and light-curing for 20 s (Cromalux LED 1200, 1400 mW/cm^2^, Rastaat, Germany).
- **CR restoration (*n* = 74)**: The enamel tissue surrounding the NCCL was etched selectively with a 37% phosphoric acid for 15 s. Then, the adhesive resin (Prime & Bond Elect One, Dentsply DeTrey Konstanz, Germany) was applied according to manufacturer’s instructions. The adhesive was left untouched for 20 s and air-dried for 5 s for solvent evaporation. The adhesive was light-cured for 10 s using the curing light. The CR (Ceram.X One Universal) was inserted incrementally and light-cured for 20 s. The finishing and polishing of the restoration were performed in the same way as previously described.

### Clinical examination

Two experienced and calibrated investigators except operator blindly evaluated the restorations after 1 week (baseline) and 12, 24, 36 and 60 months for the following criteria; retention, marginal discoloration, marginal adaptation, secondary caries and postoperative sensitivity according to the modified USPHS criteria [35]. Color photographs were taken at each recall. In case of disagreement, the third investigator was involved and consensus was reached. In case of restoration failure, the restoration was renewed and removed from the study but for the other restorations, no intervention was done including refurbishment or polishing.

### Statistical analysis

SPSS 21.0 was used for analysis. Dropped out patients were removed from the study. Additionally failed restorations were recorded as “Charlie” and removed from the study but were included in the analyses. The distribution of the data was examined by using the Kolmogorov–Smirnov test. Pearson’s Chi-Square and Fisher’s Exact tests were used for the comparison of the two restorative materials for each criterion. The changes across the five time points (baseline, 12, 24, 36 and 60 months) were analyzed using Cochran Q test. Pearson’s Chi-Square test was used for analysing the differences in the dimensions of the lesion and retention. The differences in the dimensions of the lesion and marginal adaptation and marginal discoloration were tested using Kruskal–Wallis H test. Pearson’s Chi-Square and Fisher’s Exact tests were used to evaluate differences/relationships between the TWI and GI and retention and marginal adaptation and marginal discoloration. Kaplan- Meier analysis was performed to compare the survival rates of the restorations. Stuart-Kendall’s Tau-c was used as a correlation coefficient. The significance level was set at *p* = 0.05.

## Results

Tables 3 and 4 summarize the demographic information of the patients and characteristics of NCCLs. Eight patients received 8, 8 patients received 6 and 9 patients received 4 NCCLs. There were no statistically significant differences between the groups in terms of lesion characteristics (angle, shape and depth) (*p* > 0.05).

**Table 3:** Characteristics of the patients

	*n*	%
Patient age range*		
40–50	10	40.00
51–60	8	32.0
> 61	7	28.0
Sex		
Female	14	56.0
Male	11	44.0

*$\overline{X }$±SD = 55.0 ± 8.3, min–max = 42–72

**Table 4:** Characteristics of the NCCLs including their internal angle, Tooth Wear Index (TWI) and Gingival Index (GI) characteristics, dimensions, and preoperative sensitivity

Score												
	0		1		2		3		4		Total	
	*n*	%	*n*	%	*n*	%	*n*	%	*n*	%	*n*	%
Internal angle*												
Maxilla												
Incisors	-	-	4	27	6	4.1	5	3.4	2	1.4	17	11.5
Canine	-	-	1	0.7	7	4.7	1	0.7	0		9	6.1
Premolar	-	-	18	12.2	19	12.8	4	2.7	0		41	27.7
Molar	-	-	5	3.4	3	2.0	-	-	0		8	5.4
Mandible												
Incisors	-	-	-	-	4	2.7	4	2.7	0		8	5.4
Canine	-	-	1	0.7	3	2.0	1	0.7	0		5	3.4
Premolar	-	-	19	12.8	21	14.2	7	4.7	2	1.4	49	33.1
Molar	-	-	4	2.7	6	4.1	1	0.7	0		11	7.4
Angle *												
CR			22	14.9	37	25.0	13	8.8	2	1.4	74	50.0
GH			30	20.3	32	21.6	10	6.8	2	1.4	74	50.0
Total			52	35.1	69	46.6	23	15.5	4	2.7	148	100
TWI**												
Maxilla												
Incisors	6	4.1	5	3.4	6	4.1	-	-	-	-	17	11.7
Canine	3	2.1	3	2.1	2	1.4	1	0.7	-	-	9	6.2
Premolar	12	8.3	14	9.7	11	7.6	2	1.4	1	0,7	40	27.6
Molar	3	2.1	2	1.4	3	2.1	-	-	-	-	8	5.5
Mandible												
Incisors	-	-	3	2.1	1	0.7	4	2.8	-	-	8	5.5
Canine	2	1,4	2	1.4	1	0.7	-	-	-	-	5	3.4
Premolar	21	14,5	22	15.2	4	2.8	-	-	-	-	47	32.4
Molar	6	4,1	2	1.4	3	2.1	-	-	-	-	11	7.6
G I ***												
	100	67.6	48	32.4	-	-	-	-	-	-	148	100
Shapes of the lesions****												
CR			43	29.1	14	9.5	17	11.5				
GH			51	34.5	11	7.4	12	8.1				
Total			94	63.5	25	16.9	29	19.6				
Dimensions of the NCCLs												
				Min-max			Median			Mean ± SD		
Depth				0.5-3.0			1.000			1.252 ± . 0477		
Cervico-incisal height				1.5-5.5			3.000			2.840 ± . 0742		
Mesio-distal width				1.5-8.5			3.500			4.090 ± . 1175		
Pre-operative sensitivity				N			%					
Yes				61			41.2					
No				87			58.8					

^*^ 1: 0–45°, 2: 45-90o, 3:90–135°, 4: 135–180°

^**^ 0: No loss of enamel surface characteristics, 1: Loss of enamel surface characteristics, 2: Loss of enamel exposing dentine for less than one third of surface, 3: Loss of enamel exposing dentine for more than one third of surface, 4: Complete enamel loss–pulp exposure secondary dentine exposure

^***^0: No bleeding, 1: Bleeding some seconds after probing, 2: Bleeding immediately after probing, 3: Bleeding on probing spreading towards the marginal gingiva

^****^ 1: Wedge, 2:Sharp, 3: Saucer or rounded

    The recall rates at baseline and 12, 24, 36 and 60 months were 100%, 88.0% 88.0%, 60% and 60% respectively. The distribution of the USPHS scores of the restorations at each recall is presented in Table 5.

**Table 5:** Modified USPHS evaluations for restorations at baseline, 12, 24, 36 and 60 months

Groups										
Category	GH					CR				
	Baseline *n* (%)	12-Month *n* (%)	24-Month *n* (%)	36-Month *n* (%)	60-Month *n* (%)	Baseline *n* (%)	12-Month *n* (%)	24-Month *n* (%)	36-Month *n* (%)	60-Month *n* (%)
Retention										
Alpha	74/74 (100)	57/63 (90.5)	53/63 (84.1)	32/48 (66.7)	32/48 (66.7)	74/74 (100)	58/63 (92.1)	57/63 (90.5)	38/49 (77.6)	36/49 (73.5)
Charlie	-	6/63 (9.5)	10/63 (15.9)	16/48 (33.3)	16/48 (33.3)	-	5/63 (7.9)	6/63 (9.5)	11/49 (22.4)	13/49 (26.5)
Marginal adaptation										
Alpha	74/74 (100)	50/57 (87.7)	37/53 (69.8)	28/32 (87.5)	25/32 (78.1)	74/74 (100)	55/58 (94.8)	53/57 (93.0)	33/38 (86.8)	30/36 (83.3)
Bravo	-	7/57 (12.3)	16/53 (30.2)	4/32 (12.5)	6/32 (18.8)	-	3/58 (5.2)	4/57 (7.0)	5/38 (13.2)	5/36 (13.9)
Charlie	-	-	-	-	1/32 (3.1)	-	-	–	-	1/36 (2.8)
Marginal discoloration										
Alpha	74/74 (100)	54/57 (94.7)	49/53 (92.5)	21/32 (65.6)	8/32 (25.0)	74/74 (100)	53/58 (91.4)	50/57 (87.7)	25/38 (65.8)	15/36 (41.7)
Bravo	-	3/57 (5.3)	4/53 (7.5)	11/32 (34.4)	16/32 (50.0)	-	5/58 (8.6)	7/57 (12.3)	12/38 (31.6)	16/36 (44.4)
Charlie	-	-	-	-	8/32 (25.0)	-	-	-	1/38 (2.6)	5/36 (13.9)
Secondary caries										
Alpha	74/74 (100)	57/57 (100)	53/53 (100)	32/32 (100)	32/32 (100)	74/74 (100)	58/58 (100)	57/57 (100)	38/38 (100)	36/36 (100)
Charlie	-	-	-	-	-	-	-	-	-	-
-Post-operative sensitivity										
Alpha	74/74 (100)	57/57 (100)	53/53 (100)	32/32 (100)	32/32 (100)	74/74 (100)	58/58 (100)	57/57 (100)	38/38 (100)	36/36 (100)
Charlie	-	-	-	-	-	-	-	-	-	-

At 12-month recall, 3 patients were lost (1 patient changed city and 2 patients did not answer the calls for invitation). Ten patients (2 patients stated that they received full-crowns, 2 patients stated that they moved to another city, 3 patients stated that they did not like to attend recalls due to Covid-19 pandemic and 3 patients did not answer the invitation calls) did not attend 36 months recalls. Ninety-seven restorations at 15 patients were evaluated at the 36- month recall. Forty-eight-month recall could not be done due to Covid-19 pandemic. At the 60-month recall, all 15 patients (with 97 restorations) who had attended the 36-month recall were present for re-evaluation.

**Fig. 2:**
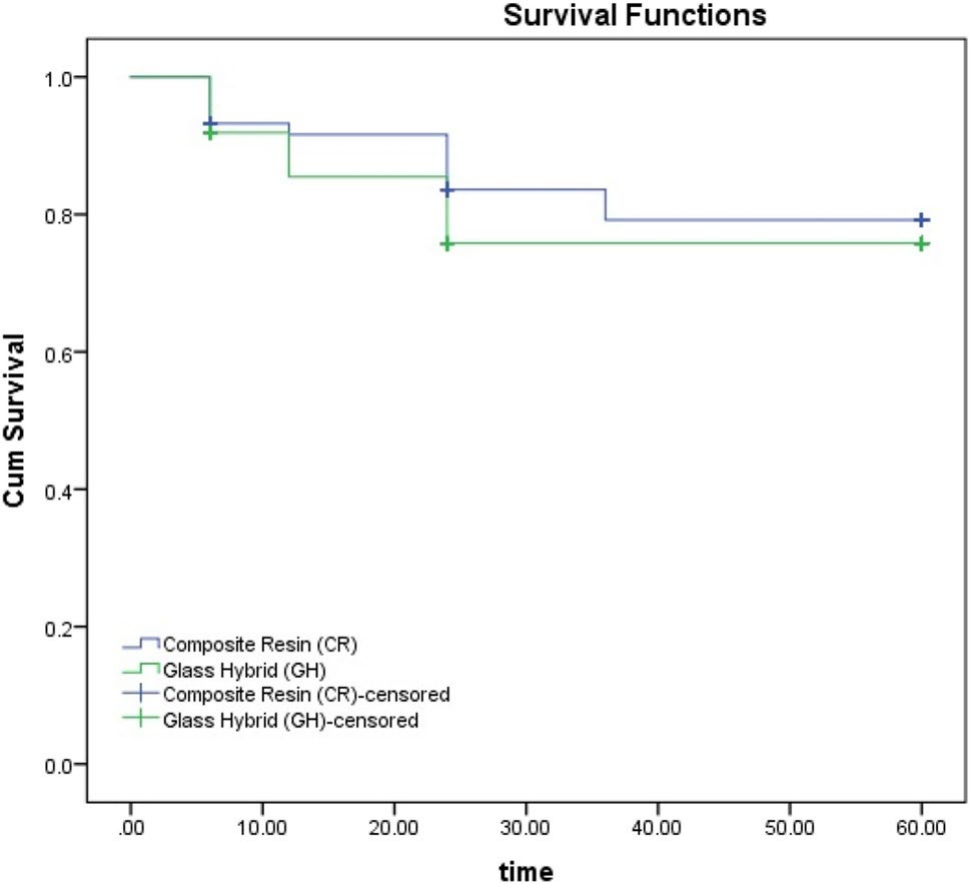
Kaplan–Meier curve analysis comparing groups

After 12 months, 6 GH /5 CR (11) restorations; after 24 months, 4 GH / 1 CR (5) restorations and after 36 months, 6 GH/ 5 CR (11) restorations failed. At 60- month recall, 2 CR restorations failed. After 60 months, 29 restorations (13 CR, 16 GH) lost retention. Retention rates were 90.5%, 84.1%, 66.7% and 66.7% for GH and 92.1%, 90.5%, 77.6% and 73.5 for CR restorations at 12, 24, 36 and 60 months, respectively. There was no significant difference between the restorative materials in terms of retention at 12, 24, 36 and 60 months (*p* = 0.752, *p* = 0.285, *p* = 0.232, *p* = 464, respectively). Kaplan–Meier analysis with log rank test revealed no difference between the materials (*p* = 0.487) (Fig. 2).

The relationship between the materials and retention, internal angle, depth, cervico-incisal height, mesio-distal width of the lesions, TWI and GI is presented in Table 6. A statistically significant relationship was observed between CR’s retention and TWI at all evaluation times. Cochran Q test revealed a significant change over time in terms of retention in both groups (*p* < 0.001, *p* < 0.001). There was a moderate and negative relationship between TWI and retention at 36 and 60-months (Stuart-Kendall’s Tau c = -0.351, -0.420, respectively).

**Table 6:** The relationship between the restorative materials and retention / internal angle / depth / cervico-incisal height / mesio-distal width of the lesions / TWI / gingival index (*p* values*)

	GH			CR		
	Retention	Marginal adaptation	Marginal discoloration	Retention	Marginal adaptation	Marginal discoloration
12- Month						
Height	0.187	0.979	0.230	0.138	0.426	0.823
Width	0.610	0.234	0.132	0.524	0.686	0.381
Depth	0.339	0.594	0.863	0.168	0.075	0.272
Internal angle**	0.293	0.221	0.441	0.792	0.446	0.316
TWI***	0.917	**0.011**	0.342	**0.010**	**0.031**	0.469
GI****	**0.037**	**0.013**	0.116	0.866	0.180	0.854
24- Month						
Height	0.801	0.349	0.842	0.704	**0.002**	0.989
Width	0.907	0.415	0.660	0.979	0.927	0.327
Depth	0.357	0.210	0.939	0.179	0.983	0.314
Internal angle**	0.281	0.712	0.639	0.155	0.917	0.383
TWI***	0.956	** < 0.001**	0.390	**0.038**	0.094	0.590
GI****	**0.036**	0.002	0.058	0.865	0.149	0.514
36- Month						
Height	0.440	0.482	0.456	0.44	0.482	0.456
Width	0.342	0.985	0.880	0.342	0.985	0.880
Depth	0.721	0.071	0.842	0.721	0.071	0.842
Internal angle**	0.576	0.782	0.600	0.576	0.782	0.600
TWI***	**0.002**	0.078	0.330	**0.002**	0.078	0.330
GI****	0.766	0.550	0.050	0.766	0.550	0.050
60- Month						
Height	0.709	0.422	**0.038**	0.007	0.841	0.629
Width	0.245	0.410	0.811	0.093	0.430	0.871
Depth	0.142	0.597	0.870	0.491	0.423	0.954
Internal angle**	0.095	0.908	0.344	0.428	**0.050**	**0.044**
TWI***	0.107	**0.035**	0.315	**0.002**	0.270	0.445
GI****	**0.030**	0.646	0.319	0.866	0.098	0.180

^*^Mann–Whitney U test, Kruskal–Wallis H test results

(Bold values indicate statistical significance.)

^*^ *1: 0–45°, 2: 45-90o, 3:90–135°, 4: 135–180°

^***^ 0: No loss of enamel surface characteristics, 1: Loss of enamel surface characteristics, 2: Loss of enamel exposing dentine for less than one third of

surface, 3: Loss of enamel exposing dentine for more than one third of surface, 4: Complete enamel loss–pulp exposure secondary dentine exposure

^****^ 0: No bleeding, 1: Bleeding some seconds after probing, 2: Bleeding immediately after probing, 3: Bleeding on probing spreading towards the marginal gingiva

At 12-month recall, 10 (7 GH and 3 CR); at 24-month recall 20 (16 GH and 4 CR); at 36-month recall 9 (4 GH, 5 CR); at 60-month recall 11 (6 GH, 5 CR) restorations were scored as Bravo and 2 restorations (1 GH, 1 CR) were scored as Charlie for marginal adaptation (Table 2). There was a significant difference between the restorative materials at 24- month recall (*p* = 0.002) but there were not after 36- and 60- month recalls (*p* = 0.935 and *p* = 0.856, respectively), since the dropouts at 36- month recall primarily consisted of previously Bravo scored restorations regarding marginal adaptation. Cochran’s Q tests revealed a significant change in terms of marginal adaptation over time in both groups up to 60 months (*p* < 0.001, *p* < 0.001).

At 12-month recall, 8 (3 GH and 5 CR); at 24-month recall 11 (4 GH and 7 CR) and at 36-month recall 23 restorations (11GH and 12 CR) were scored as Bravo and 1 CR restoration was scored as Charlie in terms of marginal discoloration. At 60-month recall, 32 (16 GH and 16 CR) restorations were scored as Bravo and 13 restorations (8 GH and 5 CR) were scored as Charlie. Both materials showed significant (Cochran’s Q test, *p* < 0.001), but similar levels of marginal discoloration at 12, 24, 36, and 60 months (*p* = 0.479, *p* = 0.408, *p* = 0.999, and *p* = 0.273, respectively) (*p* < 0.001). Tooth sensitivity or secondary caries were not observed at any time points evaluated (Table 4).

## Discussion

The real performances of restorative materials can only be assessed adequately with clinical studies. Due to the presence of sclerosed dentin, lack of retentive areas and direct occlusal stresses, NCCLs are the most commonly used tooth regions for evaluating the retention performances of these materials [8, 22]. In order to eliminate the confounding cervical stresses generated in NCCLs, bruxism is generally viewed as a criterion for exclusion in clinical trials [23, 35, 36]. However, this exclusion reflects the outcomes of an unrealistic estimation since there is a direct correlation with NCCL and bruxism in real life [37]. Thus, the present randomised, split-mouth clinical study was specifically planned to assess the most realistic clinical performances of two different restorative materials in patients with bruxism. At the end of 60-month evaluation period, the null hyothesis was accepted since, no significant difference was found between the tested materials regarding the criteria assessed at any time.

The retention is the most important criterion when evaluating the clinical performance of a restoration. It was commonly reported that GIC restorations showed higher retention rates than CRs in NCCLs [38]. It was previously reported that the presence of sclerotic dentin in NCCLs causes the obliteration of dentinal tubules which leads to weak adhesion of CRs, but the micromechanical adhesion mechanism can still form adequate bond strength [38–41]. Besides, low elasticity modulus of GICs, presents them as the suitable choice for areas where occlusal forces concentrate [38, 42]. However, the results of the present study showed that, after 60 months follow-up, 29 restorations were lost in both groups (13 CR (26.5%), 16 GH (33.3%)). Although fewer failures were seen with CR, no significant difference was observed between the retention rates of the materials (73.5% for CR and 66.7% for GH). Similarly, Uzer-Çelik et al. [43] compared the GH, which they named high viscosity glass ionomer cement (HVGIC) with a CR in the restoration of NCCLs and reported higher retention rates with the CR at 36-month recall. In another study, resin-modified glass ionomer cement (RMGIC), HVGIC and nanohybrid CR restorations in NCCLs were evaluated and after 1 year and RMGIC and HVGIC showed significantly higher retention rates than the nanohybrid CR [44]. The authors attributed this result to the presence of calcified dentinal tissue caused by the inclusion of mostly elder patients in the study group. Schwendicke et al. [45] also compared the survival, restoration quality and costs of GH and CR restorations of sclerotic NCCLs after 3 years. At the end of 3 years, the survival of GH restorations was not significantly different; however, was less costly both initially and in long-term than CR for restoring NCCLs. It is reported that GIC materials form more reliable bonds with sclerotic dentinal tissues, owing to their chemical bonding capabilities when compared to adhesive resins [46]. The only exception to this comparison would be adhesives that contain 10-MDP [45]. Besides the benefit of chemical adhesion, the noted lower retention rate of GH (though not statistically significant) may be linked to the bulk application of the GH material. This technique could potentially affect the adaptability of restorative material to the cavity walls: a speculation also echoed by Uzer Çelik et al. [47]. The possibility of imperfect adaptation of the restorative material, when combined with excessive occlusal loads of bruxism, might have lead to the loss of GH restorations more than expected. All in all, no significant difference was found between the retention rate of the materials. Therefore, as concluded in a previous study, both GH and CR exhibited promising results for the restoring NCCLs in patients suffering from bruxism [42] and GH restorative has an additional advantage of low cost-effectiveness [48, 49].

In this study, no cavity preparation such as enamel bevelling or dentin roughening with burs were performed, in line with previous studies [50, 16, 51, 52]. This was done to preserve healthy tooth tissue and to standardize the conditions for both materials, given that enamel bevelling is not advised for GICs [53]. It was reported that enamel beveling did not positively affect the retention rates of NCCL restorations [54, 55]. However, roughening dentin surfaces was associated with improvement in retention rates of NCCL restorations due to the removal of sclerotic layer [56–58]. While the absence of dentin roughening could potentially compromise the bonding of CR, in this study; it's worth noting that universal adhesives are frequently used in NCCLs without dentin roughening [50, 52, 16, 59, 60], owing to their mechanical adhesion capability provided by MDP [45]. Ultimately, this remains speculative, as to the best of the authors' knowledge, there are no clinical data on the impact of dentin roughening on the retention rates of universal adhesives.

Viewing the retention results of this study from another perspective, the retention rates of the current study (66% for GH and 73.5% for CR) were lower than those reported by Schwendicke et al. [45] (73% for GH and 79% for CR). In their study, bruxism wasn't considered as an exclusion criterion, suggesting that some of their participants might also have been bruxists like those in the present study. While it is possible to speculate that bruxism may have contributed to the lower retention rates in the current study, a direct comparison between these studies remains unfeasible due to the clustered-parallel nature of their study, their low recall rate and their lack of detailed reporting on the quantitative aspects of bruxism presence. Furthermore, when compared to the results of Uzer-Çelik et al. [47]( 87% for GH and 100% for CR), the retention rates of the present study were notably lower. This discrepancy might also be attributed to the inclusion of bruxist patients in the current study. Although, the results of the present study indicated a potential correlation between bruxism and higher retention loss in NCCL restorations; more detailed and focused studies are needed to confirm this correlation and to further explore the mechanisms by which bruxism could influence the retention rates of NCCL restorations.

The marginal adaptation and discoloration bother patients and clinicans due to aesthetics and anatomic form. The insufficiency of enamel tissue in the NCCLs may contribute to deficient adaptation of the dental materials and discoloration of the margins [61]. Additionally, the color stability of resin based restorative materials is mainly influenced by adequate polymerization, since residual monomers undergo sorption of oral fluids and results in discoloration [62, 63]. CRs have a more homogeneous surface due to presence of filler particles, nevertheless inadequate polymerization of the resin matrix makes them vulnerable to discoloration [62]. Although the GH restorative used in this study doesn’t contain resin in its structure, a resin coating agent was applied subsequent to the finishing and polishing procedure following the directions of the manufacturer. The findings of the present study revealed no significant difference between the tested materials for marginal adaptation and discoloration at the end of 60 months. Both materials showed moderate and significant changes with time for these criteria. However, the slight deteriorations observed over time were still in the clinically acceptable range. The alterations on marginal adaptation and discoloration might be related to the small chippings on the restoration margins, wear of the coating material on GH restorative and sorption of the oral fluids by the CR. This study presented results in line with the meta-analysis report of Bezerre et al. [19], which concluded no significant difference between CR and GIC for marginal adaptation and discoloration. On the other hand, since high viscosity GH restorative materials diverse from former GICs with changes in their chemical and mechanical properties and there has been no published long-term clinical study about the performance of current GH restorative, it is impossible to completely interpret the results of this study comparing to others. Additionally, it's crucial to note that the study dropouts at 36- month recall primarily consisted of restorations that were previously scored as 'Bravo' for marginal adaptation at 24-month recall. This could have contributed to an artificially positive trend in marginal adaptation at the 36 and 60-month intervals. Therefore, it's plausible to speculate that over time, the variations in marginal adaptation might have been significant if not for these dropouts.

The challenges clinicians face when restoring NCCLs have urged many researchers to conduct numerous studies regarding the factors that may affect the survival of these restorations. On this respect, internal angles, dimensions, severity and characteristics of NCCLs and the presence of inflammation in neighbouring gingival tissues were reported to influence the success of restorations [64–66]. Thus, in the present study, the influence of these factors on retention, marginal adaptation and marginal discoloration were also evaluated and the recent findings revealed that TWI can alter the clinical performance of the tested CR material. TWI, being a clinical measurement of tooth wear can give insight related to the presence of dentin sclerosis. Supporting the results of the present study, it was commonly reported that the presence of sclerotic dentin in NCCLs causes the obliteration of dentinal tubules which leads to weak adhesion of CR, but the micromechanical adhesion mechanism can still form adequate bond strength [19]. This might explain the significant relationship between TWI and failures in CR whereas there was no significant relation between the retention rates of GH and TWI. Regarding the marginal adaptation; a moderate, positive and significant relationship between marginal adaptation and TWI across all time points was observed except the 36-month interval. The lack of this relationship at 36 months can likely be attributed to artificially elevated marginal adaptation scores due to the dropouts as previously mentioned.

This clinical study had some limitations just like other clinical trials. The study was conducted in a university setting and all the restorative procedures were performed by a well-calibrated and experienced operator (UKV). The clinical outcomes may vary in other healthcare institutions providing dental services and with the experience of the operators. Furthermore, the inclusion of only bruxist patients makes it impossible to make a clear comparison about the performances of these materials with previous studies. Another important point is the technique used for isolation. Considering a previous meta-analysis study which stated that rubber dam isolation was not an indispensable requirement for long-lasting restorations if sufficient isolation can be achieved with other techniques [61], cotton roll isolation was preferred in this study in order to project the outcomes of a more frequently used isolation method in daily practice. Additionally, no blinding of the operator was possible due to the marked differences in the application protocols of the tested restorative materials and more than one pair of restorations were placed on some patients which might affect the outcomes due to individual factors like bruxism severity and characteristics of fluctuations. Besides, patients with self-reported bruxism and clinical signs of bruxism were included but polysomnography was not done. Likewise, the present-minded bruxism activity of the patients could not be monitored between the recalls. Therefore, the follow-up period of this study should be extended further and new clinical studies in accordance with previous protocols should be conducted to overcome the current limitations.

## Conclusion

In this 60-month study, the tested GH and nano-ceramic CR restorative materials showed similar and reliable results for the restoration of NCCLs in bruxist individuals. In clinical practice, both materials can be considered reliable choices for the restoration of NCCLs in patients with bruxism, providing aesthetic and functional benefits over an extended period. Dentists should carefully assess individual patient needs and clinical conditions to make informed decisions regarding the selection of restorative materials.

## Limitations

1. Polysomnography was not used to diagnose bruxism
2. Rubber dam was not used for isolation
3. The operator was not blinded
4. More than one pair of restorations were placed on some patients
